# Cell-free DNA in newly diagnosed patients with glioblastoma – a clinical prospective feasibility study

**DOI:** 10.18632/oncotarget.27030

**Published:** 2019-07-09

**Authors:** Dorte Schou Nørøxe, Olga Østrup, Christina Westmose Yde, Lise Barlebo Ahlborn, Finn Cilius Nielsen, Signe Regner Michaelsen, Vibeke Andrée Larsen, Jane Skjøth-Rasmussen, Jannick Brennum, Petra Hamerlik, Hans Skovgaard Poulsen, Ulrik Lassen

**Affiliations:** ^1^ Department of Radiation Biology, Rigshospitalet, 2100 Copenhagen, Denmark; ^2^ Department of Oncology, Rigshospitalet, 2100 Copenhagen, Denmark; ^3^ Center for Genomic Medicine, Rigshospitalet, 2100 Copenhagen, Denmark; ^4^ Department of Neuroradiology, Rigshospitalet, 2100 Copenhagen, Denmark; ^5^ Department of Neurosurgery, Rigshospitalet, 2100 Copenhagen, Denmark; ^6^ Danish Cancer Society, 2100 Copenhagen, Denmark

**Keywords:** glioblastoma, liquid biopsy, cell-free DNA, fragment length, base-pair

## Abstract

**Background:** Glioblastoma (GB) is an incurable brain cancer with limited treatment options. The aim was to test the feasibility of using cell-free DNA (cfDNA) to support evaluation of treatment response, pseudo-progression and whether progression could be found before clinical and/or radiologic progression.

**Results:** CfDNA fluctuated during treatment with the highest levels before diagnostic surgery and at progression. An increase was seen in 3 out of 4 patients at the time of progression while no increase was seen in 3 out of 4 patients without progression. CfDNA levels could aid in 3 out of 3 questionable cases of pseudo-progression.

**Methods:** Eight newly diagnosed GB patients were included. Blood samples were collected prior to diagnosis, before start and during oncologic treatment until progression. Seven patients received concurrent radiotherapy/Temozolomide with adjuvant Temozolomide with one of the patients included in a clinical trial with either immunotherapy or placebo as add-on. One patient received radiation alone. CfDNA concentration was determined for each blood sample.

**Conclusions:** It was feasible to measure cfDNA concentration. Despite the limited cohort size, there was a good tendency between cfDNA and treatment course and -response, respectively with the highest levels at progression.

## INTRODUCTION

Glioblastoma (GB) is a highly malignant brain tumor with limited treatment options. With standard treatment, median overall survival (OS) is 16–22 months [1]. The standard method for monitoring a treatment response is by clinical evaluation of the patient and by magnetic resonance imaging (MRI) at defined intervals ranging from two to six months using Response Assessment in Neuro Oncology criteria (RANO) [2]. However, pseudo-progression is seen in approximately 20% of patients [3, 4] and can be difficult to distinguish from true progression. Only surgery with following pathological confirmation of vital tumor cells in the lesion can verify the progressive state. For cases in which only treatment related changes are found, the surgery could have been futile with valuable time lost in which a different treatment could have been effectuated. Or worse, the surgery will leave the patient clinically unfit for further treatment. Therefore, a less time consuming and non-invasive method for treatment monitoring is needed and a blood-based biopsy seems promising. Several methods exist to monitor liquid-based alterations [5–9] that includes circulating tumor cells (CTCs) or alterations detected in cerebrospinal fluid (CSF). CTCs can be detected in blood in up to 39% of GB patients [10] in experimental settings, using antibodies to target epithelial cell adhesion molecules or by detecting the malaria protein VAR2CSA which is expressed in GB-cell lines [11]. Alterations detected in CSF have been identified in 49.4% of glioma patients with neurologic symptoms [12]. Another more accessible tumor source is circulating cell-free DNA (cfDNA) and specifically circulating tumor DNA (ctDNA). CtDNA is more fragmented than normal cfDNA making size selection strategies usable to indicate tumor fraction [13–16]. The fraction of ctDNA in cancer patients accounts for 3–93% of the total cfDNA [17], thus cfDNA can be used as a surrogate marker of tumor activity/burden. The shedding of tumor DNA has been found to be increased according to tumor burden, necrosis and apoptosis but can also be caused by normal cell degradation from e.g. infection, stroke, renal failure or even strong exercise [18–21]. Elevated cfDNA levels have been detected in patients with severe brain injury which is proof-of-principle that cfDNA is shed from the brain to the blood stream during cell degradation [22, 23] and ctDNA has been detected in patients with brain cancer [24–26]. In this study, we aimed to test the feasibility of detecting cfDNA in patients with GB and to investigate if cfDNA fluctuations could support evaluation of treatment response, pseudo-progression and whether progression could be found before clinical and/or radiologic progression.

## RESULTS

### Included patients and their clinical course

A total of eight patients were included for further analyses. One patient was treated with 34 Gy/10F and seven patients received RT/TMZ with adjuvant TMZ. One of these seven patients was treated in an experimental trial with a Programmed Death1 inhibitor (PD1i) or placebo as add-on to the standard treatment. (Table 1) At time of data lock four patients had progressed and of the four patients without progression, two were still on-treatment and two were in a follow-up (FU)-program*.*

**Table 1:** Overview of included patient

Patient	Age	Gender	IDH/MGMT-status	Treatment	Pseudo-progression	Complications during study period	Progression
**GB1**	52	Male	*IDH*-WT/ MGMT-WT	RT/TMZ	Yes		Yes
**GB2**	59	Female	*IDH*-WT/ MGMT-meth	RT/TMZ	Yes	Meningitis	Yes
**GB3**	46	Male	*IDH*-WT/ MGMT-WT	RT/TMZ	No		Yes
**GB4**	77	Male	*IDH*-WT/ MGMT-meth	34 Gy/10 F	No		Yes
**GB5**	59	Female	*IDH*WT/ MGMT-WT	RT/TMZ	Yes	The patient declined further treatment after 5 cyc of adj TMZ and was taken off-study.	No. FU-program
**GB6**	62	Female	*IDH*-WT/ MGMT-meth	RT/TMZ	No	No more blood samples were drawn after two cyc of adj TMZ due to logistics and patient compliance.	No. FU-program
**GB7**	46	Male	*IDH*-WT/ MGMT-meth	RT/TMZ plus PD1i/placebo	No	Intracerebral bleeding	No, on-treatment
**GB8**	52	Male	*IDH*-WT/ MGMT-meth	RT/TMZ	No		No, on-treatment

Abbreviations: *IDH*: isocitrate-dehydrogenase; WT: wild type; MGMT: O-6-methyl-guanine-DNA-methyl-transferase; RT/TMZ: radiotherapy/Temozolomide; PD1i: programmed death1 inhibitor; Gy: grey; cyc: cycles; adj: adjuvant; FU; follow-up.

### cfDNA fluctuated during treatment with the highest value at progression

It was feasible to collect blood samples in patients with GB before, during and after planned treatment. As shown in Table 2 the mean cfDNA before surgery was 12.5 ng/ml (range 2.4–63) and dropped to 7.9 (range 0.3–26.4) one month after, just before start on RT/TMZ. The mean cfDNA then reached 8.3 ng/ml (range 4.1–13.8) at the highest individual value during RT and decreased to 4.9 ng/ml (range 1.5–6.9) after RT/TMZ just before start on adjuvant TMZ. The highest value at all time points was at progression with a mean of 23.4 ng/ml (range 2.4–73.4)*.* During RT/TMZ, a mean cfDNA at the highest individual level was 8.3 (range 4.1–13.8). During RT/TMZ, four patients had the highest concentrations after 20 Gy, one after 30 Gy and one after 40 Gy but levels were relative constant between 0.3–10.5 ng/ml except for GB1 who had a constant decrease. (Supplementary Figure 1).

**Table 2:** Mean concentration of cfDNA (ng/ml plasma) and base pair (bp)-peaks at defined intervals in the study period

Time	Number of evaluable patients	Mean cfDNA ng/ml (range)	Mean bp-peak (range)
**Before diagnostic surgery**	7	12.5 (2.4–63.0)	153 (136–171)
**One month after surgery**	7	7.9 (0.3–26.4)	147 (134–166)
**During RT (highest individual value)**	7	8.3 (4.1–13.8)	148 (138–154)
**One month after RT**	6	4.9 (1.5–6.9)	153 (147–163)
**At progression**	4	23.4 (2.4–73.4)	132 (120–144)

The number of evaluable patients at each step is shown.

### cfDNA increased before or at radiologic progression in three out of four patients

For the four patients who progressed during the study period (GB1-4), cfDNA concentration and selected MRI´s as related to treatment and time from diagnosis, is shown in Figure 2A–2E. We could detect an increase in cfDNA in GB1, day 155 and GB2, day 345 before radiologic progression. An increase at progression was seen in GB4, day 205. GB3 did not have an increase in cfDNA before radiologic progression. Unfortunately, the blood sample before progression could not be analyzed why a potential increase prior to radiologic progression could not be determined. A detailed description of all eight patients is given in Supplementary Table 1.

**Figure 1:**
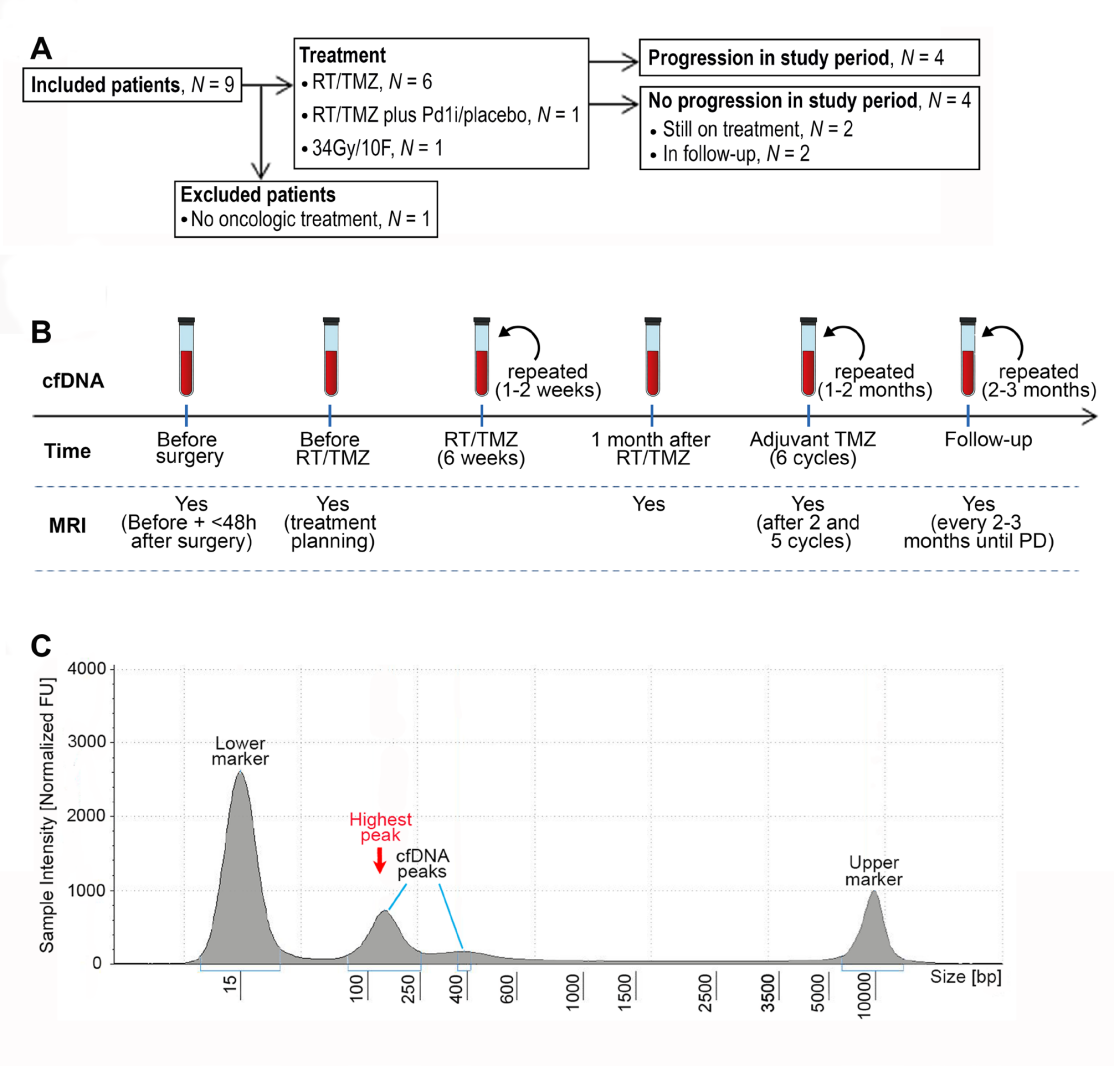
CONSORT diagram, workflow and example of fragment size analysis. (**A**) CONSORT diagram of included patients. Abbreviations: RT/TMZ: radiotherapy/Temozolomide (concurrent RT/TMZ plus adjuvant TMZ); RT/TMZ plus PD1i/placebo: (concurrent RT/TMZ plus Programmed Death1 inhibitor/placebo followed by adjuvant TMZ plus PD1i/placebo). (**B**) Illustration of work flow. First sample was taken the day before or on the day of diagnostic surgery. If the diagnosis of glioblastoma was confirmed, the next sample was taken one month after surgery at first visit to the oncologic department, every 1–2 weeks during RT/TMZ, throughout the adjuvant TMZ with 1–2 months interval and in the follow-up period with approximately 2–3 months interval until progressive disease (PD). Magnetic resonance imaging (MRI) was performed before surgery, ≤48 hours after surgery, for treatment planning of RT/TMZ, one month after RT/TMZ, after two and five cycles of adjuvant TMZ and then every 2-3 months until progression. (**C**) An example of a result from the fragment analysis assessed using the Tape Station instrument. CfDNA fragments were visualized using a higher and lower ladder as reference, respectively*.* The peak of the curve with the highest % of fragments, was defined as the highest peak.

**Figure 2:**
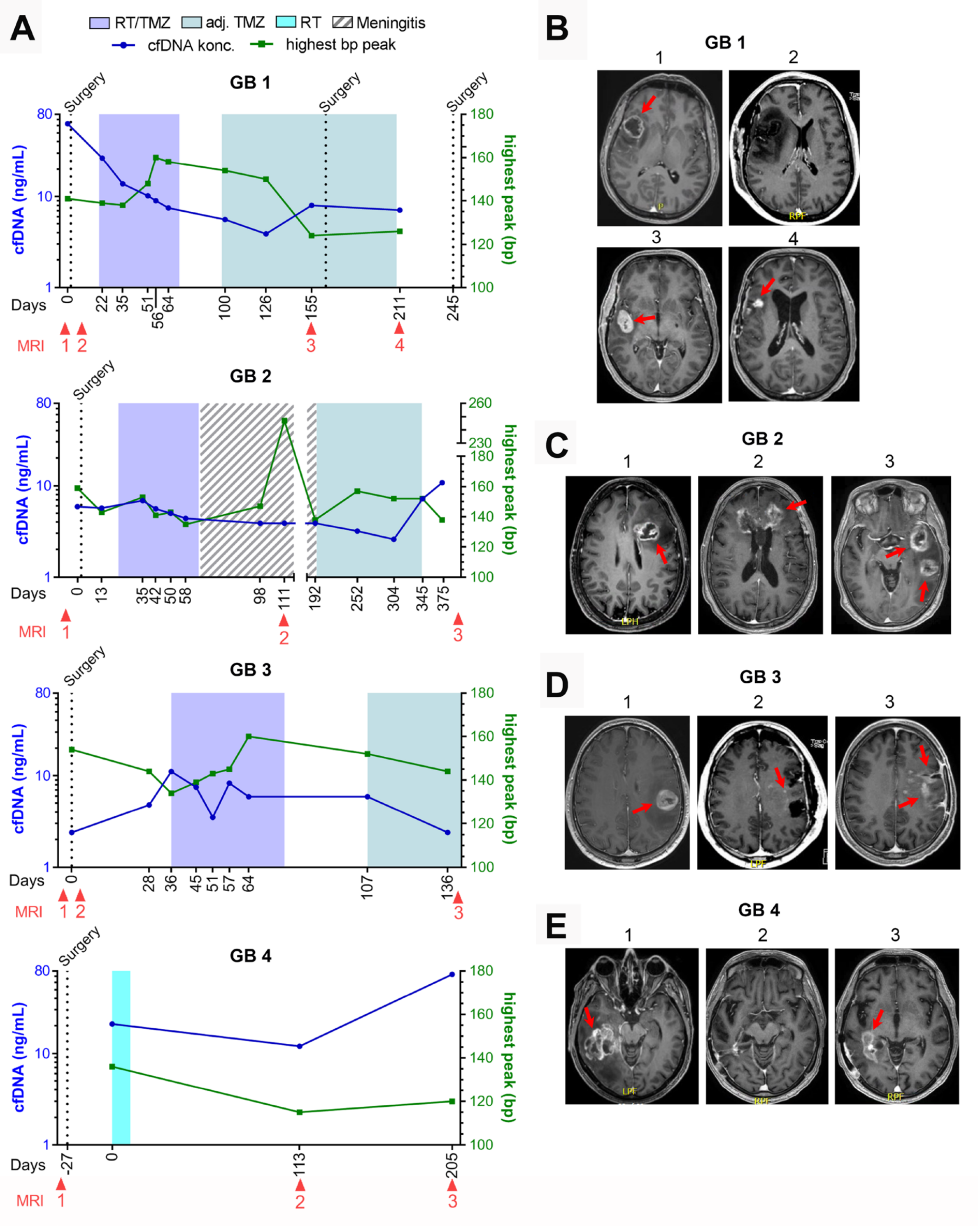
GB1-4 with progression. (**A**) Fluctuations in cell-free DNA (cfDNA) during study period at different treatment times with correlated basepar (bp) peaks. (**B**–**E**) Selected magnetic resonance imaging (MRI) during treatment period. (B) Patient GB1. B1: 30 × 29 mm contrast-enhanced (CE) tumor at diagnosis, B2: <48 hours after surgery with no residual tumor left, B3: 32 × 18 mm CE tumor, B4: progression in tumor cavity including new lesions to a total of 515 mm^3^ CE tumor. (C) Patient GB2. C1: 32 × 23 mm CE tumor at diagnosis, C2: During meningitis treatment showing growth of known CE tumor, including new lesions to a total of 2099 mm^3^ CE tumor, C3: Progression of all tumor lesions to a total 3863 mm^3^. (D) Patient GB3. D1: 28 × 27 mm CE tumor at diagnosis, D2: <48 hours after surgery showing no measurable residual tumor but two punctate CE-lesions, D3: Progression to a 21 × 12 mm CE tumor and new non-measurable lesions. (E) Patient GB4. E1: 49 × 39 mm CE tumor at diagnosis. E2: No measurable CE tumor. E3: Progression with a 62 × 20 mm CE tumor.

### cfDNA did not increase in three out of four patients without progression

**Figure 3:**
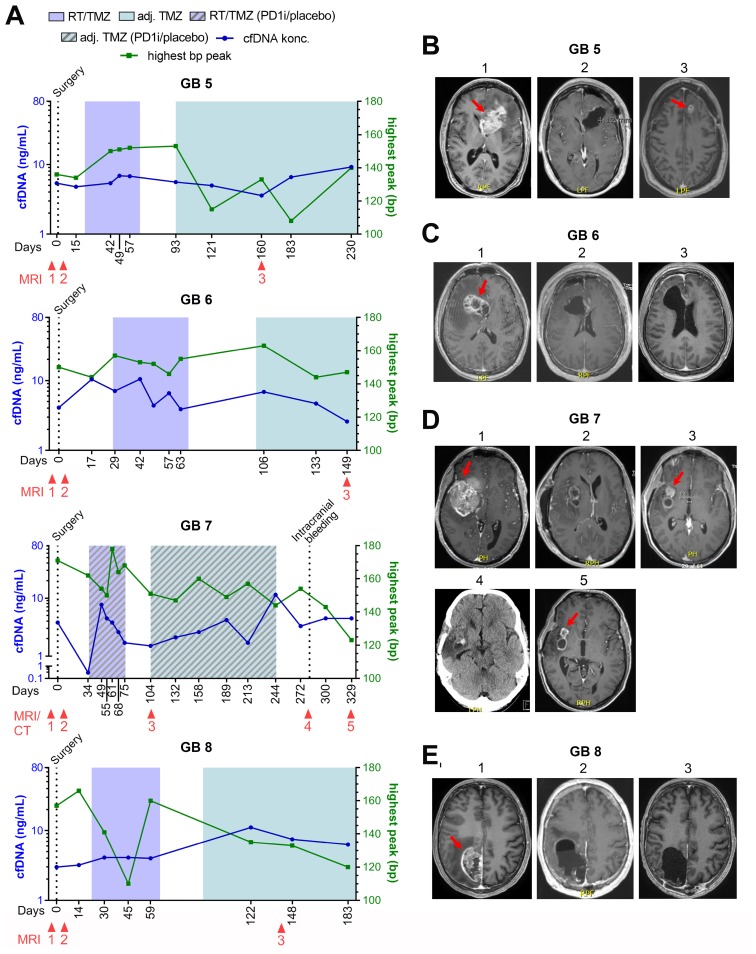
GB5-8 without progression. (**A**) Fluctuations in cell-free DNA (cfDNA) during study period at different treatment times with correlated base pair (bp) peaks. (**B**–**E**) Selected magnetic resonance imaging (MRI) during treatment period. (B) Patient GB5. B1: 65x36 mm contrast-enhanced (CE) tumor at diagnosis, B2: <48 hours after surgery with no residual tumor, B3: 17 × 12 mm CE tumor (pseudo-progression). (C) Patient GB6. C1: 46 × 29 mm CE tumor at diagnosis, <48 hours after surgery showing a small non-measurable CE lesion, C3: stable disease with non-measurable CE lesion. (D) Patient GB7. D1: 69 × 47 mm CE tumor at diagnosis, D2: <48 hours after surgery showing residual CE tumor of 1040 mm^3^, D3: Regression to 20 × 16 mm CE tumor, D4: A computed tomography (CT) scan showing an intracerebral bleeding in the tumor cavity, D5: Further regression to a total of 243 mm^3^ CE tumor. (E) GB8. E1: 56 × 33 mm CE tumor at diagnosis, E2: <48 hours after surgery with no residual tumor, E3: Stable disease with no tumor.

For the four patients who did not progress during the study period (GB5-8), cfDNA concentration, selected MRI´s and one computed tomography (CT)-scan as related to treatment and time from diagnosis, is shown in Figure 3A–3E. At time of data-lock, GB5-6 were in a FU-program and GB7-8 were still on-treatment. No increase in the latest cfDNA concentrations were noted in GB6-8. GB5 had an increase in the latest measurements and will be further discussed below.

### Pseudo-progression was supported by cfDNA in three out of three patients

Three patients were suspected of pseudo-progression based on MRI, one of whom had true progression. GB1 was suspected of progression (Figure 2B, MRI 3) with a simultaneously increase in cfDNA, day 155. He was scheduled for relapse surgery showing no vital tumor cells in the specimen, but an MRI performed two months after confirmed progressive disease in the tumor cavity (Figure 2B, MRI 4). GB2 was suspected of progression (MRI not shown) with a small decrease in cfDNA at day 252 but progression was found unlikely at the multidisciplinary conference. CfDNA levels later increased on day 345 and 375 before progression was seen on MRI (Figure 2A and 2C, MRI 3). GB5 was suspected of progression day 160 based on an MRI with a stable cfDNA concentration (Figure 3A and 3B, MRI 3). It was interpreted as pseudo-progression and all three cases illustrate a potential connection between pseudo-progression and cfDNA concentration.

### Bp-peaks and clinical course

All samples but four (5%) had corresponding bp-peaks of ≤ 166 (Figures 2A and 3A). Of the four samples with bp-peak > 166, three of the measurements were in patient GB7; one before diagnosis with a 69 × 47 mm large, partly necrotic/apoptotic tumor (Figure 3D, MRI 1) and two during RT/TMZ plus PD1i/placebo. Due to tumor size and -location, he had only partial resection done at diagnosis with tumor and necrotic/apoptotic tissue left in the brain (Figure 3D, MRI 2). Concerning the higher levels during RT, a higher mean bp-peak of 150 (110–178) was seen across all patient samples during the RT as compared to the samples taken during the adjuvant setting of 139 (108–160). The last sample with bp-peak > 166 was observed in patient GB2 after RT/TMZ (Figure 2A) at the time she was diagnosed with meningitis and treated with high dose antibiotics intravenously for three months before she could start on the adjuvant TMZ. Majority of bp-analyses came out with two peaks; one with a low and high bp-fragment size distribution, respectively (Figure 1C). The curve with the highest percentage of measured fragment sizes, were in all cases the short fragment size distribution with a median bp-peak of 147 (108–247) and the curve with a smaller percentage of measured fragment size distribution, had a median bp-peak of 371 (268–2954). A calculated ratio between cfDNA and bp-peaks is shown in Supplementary Figure 2 *.*

### cfDNA did not correlate with tumor size

A table and simple scatter plot of tumor size and corresponding cfDNA concentrations is shown in Supplementary Table 2 and Supplementary Figure 3. We had 31 paired measurements, excluding one during meningitis in GB2. We did not find a correlation between tumor size and cfDNA when performing a Spearman´s correlation analysis.

## DISCUSSION

We have shown that sequential monitoring of cfDNA-levels in blood samples before diagnosis of GB and during treatment until progression is feasible and detectable. At our institution, the patients arrive at the hospital the night before surgery, leaving a limited time frame for information, collection of the informed consent and the blood sample prior to surgery in a situation where the patients were especially vulnerable due to upcoming high-risk surgery and without a diagnosis. In addition, the blood sampling had to be performed at our institution due to sample preservation and processing. These logistic matters complicated the setup. We observed that cfDNA concentrations fluctuated during treatment with the second highest mean level before diagnosis and the highest at progression. We would expect a high cfDNA concentration before diagnosis due to disruption of the blood brain barrier in combination with high tumor burden and hence shedding of tumor cells in the circulation. As expected, the mean cfDNA concentration decreased one month after surgery due to surgical removal of the tumor burden [21]. The stable levels or increase in cfDNA during RT can be caused by tumor and normal tissue necrosis as was also shown in a recent study with non-small-cell lung cancer (NSCLC) patients [27]. Approximately one month after RT and with no other interventions, the mean level decreased again which could be due to decrease of RT-induced edema and inflammation. In three out of four patients with progression, we saw an increase from the previous sample before or at radiologic progression and the opposite was the case with three out of four patients without progression and without an increase in their latest measurements. In all three questionable cases of pseudo-progression, cfDNA levels could potentially aid in deciding whether it was true progression or not. These findings have potential clinical impact since selected MRI´s in the FU period might be replaced with cfDNA, or the information could aid in determining whether a patient should undergo relapse surgery or not. Due to a small number of patients in this cohort, larger studies are needed to clarify this potential. The corresponding bp-peak of ≤ 166 in all samples but four, suggests that the monitored DNA can include tumor-specific DNA, but this has not been verified in the present study, e.g. by mutation specific sequencing, and is only hypothesis generating. It has been shown that selecting short DNA fragments can increase the fraction of ctDNA however a standardized procedure does not exist [16]. A study in hepatocellular carcinoma defined a cut-off for primarily tumor origin at < 166 bp and a negative correlation between tumor DNA and bp> 180 [28]. Others found different fragment lengths according to different tumor types with bp-length between 134–144 for GB xenografts, 110–140 for melanoma, a bp-peak at 277 in lung cancer or bp-length <100 in advanced colorectal cancer together with an equal distribution of tumor and normal cell DNA between 100–150, respectively [13, 14]. There is a distinct difference between the detection levels for e.g. colorectal- and brain cancer [29] and a ctDNA cut-off has not been defined for brain cancer patients. We found that other factors like RT, infection and necrosis/apoptosis may influence fragment size distribution as has also been shown in other studies [14, 17, 30]. We did not find a correlation between tumor size and cfDNA concentrations in our study even though we had 31 paired measurements. Tumor size was defined using CE, measurable lesions, but non-CE, non-measurable lesions can also shed cfDNA. We did not standardize time of sampling but since ctDNA has a half-life of minutes to 2.5 hours [31–33], the optimal time of sampling needs to be investigated further. Several studies have shown that increased levels of a specific mutation in the blood can be found significantly earlier than a radiologic or clinical progression [34–36] and *IDH* R132H mutation, *TERT* promotor mutation, and MGMT promotor methylation has been detected in brain cancer [24–26]. Therefore, to develop the technique further, it would be meaningful to perform targeted sequencing in plasma for specific mutations found in each patient´s tumor in a personalized strategy.

## MATERIALS AND METHODS

### Patients

Patients were screened during clinical working hours by one neuro surgeon from her outpatient clinic. The only screening criteria were suspicion of GB with subsequent diagnostic confirmation and eligibility for maximum safe surgery. A total of nine patients were identified and all were included at Rigshospitalet, Copenhagen University, Denmark, between November 2017 and June 2018. One patient decided not to receive further oncologic treatment after surgery and was excluded (Figure 1A). Each patient gave signed informed consent prior to diagnostic surgery. End of study was defined at progression, but each patient was followed until death or time of data lock (13.12.2018). All patients underwent surgery and standard pathological examination according to the World Health Organization (WHO) diagnostic criteria for brain tumors 2016 [37]. All included patients were diagnosed with GB, isocitrate dehydrogenase (*IDH)*-wildtype (WT), as assessed by Multiplex Ligation-dependant Probe Amplification (MLPA), and five patients had a O-6-methyl-guanine-DNA-methyl-transferase (MGMT)-methylated tumor, measured by promoter methylation using a cut-off of 10%. Standard oncologic treatment after surgery was offered according to the patient´s clinical status at the first visit at Department of Oncology. Each patient had MRI performed before surgery, ≤48 hours after surgery, after two and five cycles of adjuvant TMZ and then every three months until progression. If a patient was clinically stable but the MRI showed a possible progression, we could perform a ^18^Fluoro-*O-*(2) fluoroethyl-l-tyrosine/positron-emission-tomography (FET/PET) scan for further confirmation. The case was then discussed at a multidisciplinary meeting with neuro surgeons, -radiologists, -pathologists and -oncologists. Peripheral blood was collected prior to initial surgery, before oncologic therapy, during concurrent radiotherapy (RT)/Temozolomide (TMZ) plus adjuvant TMZ until progression (Figure 1B). The project was carried out in accordance with the Declaration of Helsinki and with approval from Ethics Committee (Journal number: H-17019401) and Danish Data Protection Agency (Journal number: RH-2017-269, I-Suite number: 05801).

### Blood sample collection, cfDNA determination and base pair detection

Peripheral blood was collected in cell-stabilizing Blood Collection Tubes (BCT; Streck Laboratories, Omaha, NE, USA). Total cfDNA was extracted from 4 ml plasma using the QIAsymphony Circulating DNA Kit (Qiagen, Hilden, Germany) according to the manufacturer’s instructions using an elution volume of 60μl. Quantification of cfDNA was performed using the dsDNA HS Assay Kit on a Qubit Fluorometer (Thermo Fisher Scientific, Waltham, MA) detecting double stranded DNA (> 10 pg/ μL) using intercalating fluorescent dyes. CfDNA fragment distribution was assessed using the Agilent 4200 TapeStation System (D5000)*.* This system uses electrophoresis to separate DNA fragments from 100–5000 bp. The peak of the curve with the highest % of fragments, was defined as the highest peak (Figure 1C). To investigate the relation between cfDNA and bp-peaks further, we calculated a ratio between the two using the formula: cfDNA/(bp-peak/100)^2^.

### Tumor size determination

A trained, senior neuro radiologist noted contrast enhanced (CE), measurable tumor of each MRI. We paired cfDNA concentration with tumor size if both were performed within 14 days of each other except for the MRI performed <48 hours after surgery which was paired with the cfDNA concentration one month after surgery without any treatment initiated.

### Limitations and strengths

Our study has several limitations. It is a small study with eight patients and results need to be validated in a larger cohort. Some blood samples were not taken or could not be analyzed due to logistic challenges and patient compliance. We measured only cfDNA and not ctDNA due to the scope of the study. It is a strength that it is a prospective study with GB, *IDH*-WT and multiple blood sampling throughout the planned treatment. All included patients completed the radiation course and seven out of eight patients moved to the adjuvant setting.

## CONCLUSIONS

We found that it was possible to detect cfDNA concentrations in patients with GB in sequential blood sampling. CfDNA concentrations increased at progression in three out of four patients but did not increase in three out of four patients without progression. CfDNA levels could potentially aid in three out of three questionable cases of pseudo-progression.

## SUPPLEMENTARY MATERIALS



## Abbreviations

GB: Glioblastoma
CTC: Circulating tumor cell
CSF: Cerebrospinal fluid
CfDNA: Cell-free DNA
CtDNA: Circulating tumor DNA
Bp: Base Pair
RT: Radio Therapy
TMZ: Temozolomide
OS: Overall survival
PFS: Progression-free survival
MRI: Magnetic resonance imaging
RANO: Response Assessment in Neuro Oncology
WHO: World Health Organization
MLPA: Multiplex Ligation-dependant Probe Amplification
IDH: Isocitrate Dehydrogenase
MGMT: O-6-methyl-guanine-DNA-methyl-transferase
TERT: Telomerase Reverse Transcriptase
FET/PET: ^18^Fluoro-*O-*(2) fluoroethyl-l-tyrosine/positron-emission-tomography
CE: Contrast Enhanced
PD1i: Programmed Death 1 inhibitor
NSCLC: Non-Small-Cell Lung Cancer
