# Supplementary material for: Cell-free DNA in newly diagnosed patients with glioblastoma – a clinical prospective feasibility study
Source: Oncotarget. 2019 Jul 9;10(43):4397–406. doi: 10.18632/oncotarget.27030 (PMC6633897; doi:10.18632/oncotarget.27030)
Supplement: Supplementary file 1 [file oncotarget-10-4397-s001.pdf]

Cell-free DNA in newly diagnosed patients with glioblastoma – a clinical prospective feasibility study

SUPPLEMENTARY MATERIALS

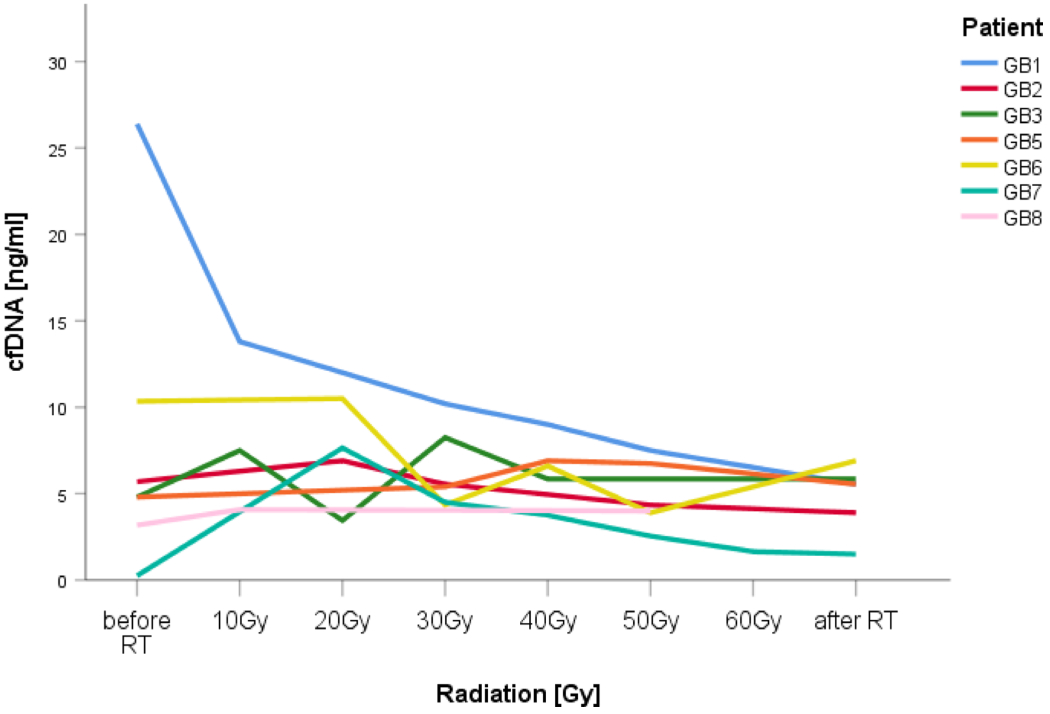

Supplementary Figure 1: Plasma samples with concentration (ng/ml) of cell free DNA (cfDNA) in seven patients throughout treatment with radiotherapy/temozolomide (RT/TMZ). GB4 received 34Gy/10F only and was not included in this figure. GB7 received RT/TMZ plus a programmed death1 inhibitor/placebo in a blinded clinical trial. Levels fluctuated with stable or increased concentrations during RT/TMZ and with stable or decrease in cfDNA four weeks after RT/TMZ. GB1 had a constant decrease throughout treatment. *Before RT* was defined as first visit at oncologic department one month after surgery without any treatment intervention and *after RT* was defined as four weeks after RT/TMZ.

Supplementary Table 1: Detailed description of included patients

Patient	Clinical description
GB1	The patient had a high cfDNA concentration before diagnosis of 63 ng/ml and one month after gross total resection, the level had decreased to 26.4 ng/ml. A continuous decrease was seen throughout the treatment course until after the second cycle of adjuvant TMZ, day 155 (Figure 1A) where cfDNA increased to 8.0 ng/ml. He was simultaneously suspected of progression based on MRI (Figure 2B, *MRI 3*) and taken to surgical theatre for re-resection. Although the histopathological examination could not detect true progression, we speculate that this patient was indeed progressing when the first increase in cfDNA was seen and base this on the fact that the patient progressed again in the surgical cavity shortly after, day 211 (Figure 2B*, MRI 4*) and was again operated with detection of vital tumor cells. CfDNA levels stayed constant during the second and third surgery (Figure 2A).
GB2	The patient had stable levels of cfDNA from diagnosis until after the fourth cycle of adjuvant TMZ. Treatment between RT/TMZ and adjuvant TMZ was interrupted by a serious incident of meningitis. After the second cycle of adjuvant TMZ, day 252 (Figure 2A), cfDNA was still stable at 3.2 ng/ml but progression was suspected based on MRI and FET/PET (not shown). Since the patient was clinically stable, it was interpreted as pseudo progression and the treatment continued. Between the fourth and fifth cycle, she was diagnosed with penia and postponed three weeks in treatment. After the fourth cycle, cfDNA increased to 7.2 ng/ml, day 345 (Figure 2A). The patient had radiologic progression approximately one month after (Figure 2C*, MRI 3*) where cfDNA had increased further to 10.8 ng/ml and she was taken off-treatment.
GB3	The patient did not have an even pattern in cfDNA fluctuation. Before diagnosis, cfDNA was measured to 2.4 ng/ml, increased to 11.1 ng/ml before start on RT/TMZ and then fluctuated between 7.5–2.4 ng/ml in the latest measurement after 1 cycle of adjuvant TMZ, day 136 (Figure 2A). He progressed after the third cycle based on MRI (Figure 2D*, MRI 3*).
GB4	The patient was treated with RT only due to age and clinical evaluation and had three blood samples drawn in total (Figure 2A). A decrease from 21 ng/ml before diagnosis to 12.0 ng/ml three months after RT was seen with stable disease on MRI (Figure 2E*, MRI 2*). CfDNA levels increased substantially to 73.4 ng/ml at progression, day 205 with confirmation on MRI (Figure 2E*, MRI 3*).
GB5	The patient had relative stable cfDNA values throughout the treatment, fluctuating between 5.4 ng/ml before diagnosis to 9.2 in the latest sample after 4 cycles of adjuvant TMZ. She was suspected of progression after the second cycle of adjuvant TMZ, day 160, (Figure 3B*, MRI 3*). At that time, cfDNA was at the lowest value of 3.6 ng/ml. Due to good clinical status, the patient continued treatment and a FU-MRI and FET/PET disproved the possible progression (not shown) but cfDNA levels started to increase to 6.6 and 9.2 ng/ml (Figure 3A), respectively after the pseudo progression, illustrating a discrepancy in elevated cfDNA levels and progression. The patient was still without evidence of progression at data lock.
GB6	The patient had relatively stable cfDNA-measurements throughout the treatment course fluctuating between 10.4 ng/ml before diagnosis to 2.6 ng/ml in the latest sample (Figure 3A). She is without evidence of progression at time of data lock (Figure 3C*, MRI 3*). Both GB5 and GB6 did not have blood samples taken since end of the adjuvant treatment; one due to patients own wish of no further treatment and one due to logistic challenges and patient compliance.
GB7	The patient had a partial resection done (Figure 3D*, MRI 1 and 2*) and cfDNA-level decreased from 3.8 ng/ml before diagnosis to 0.3 ng/ml one month after (Figure 3A). A relative stable fluctuation was then observed with regression on MRI (Figure 3D*, MRI 3*) until day 244 with a single measurement to 11.4 ng/ml. Approximately one month after, the patient was admitted to the hospital with an intracranial bleeding in the tumor cavity (Figure 3D*, CT 4*) and a subsequent MRI showed regression (not shown) why this increase could be attributed to the bleeding and not progression. The tumor continued to regress (Figure 3D*, MRI 5*) and the patient is still on-treatment at time of data lock.
GB8	The patient had stable cfDNA-levels except for one high measurement after the first adjuvant cycle of TMZ, day 122 of 11.1 ng/ml (Figure 3A), an MRI after the second cycle with stable disease (Figure 3E*, MRI 3*) and an unchanged clinical status.

Abbreviations: cfDNA: cell-free DNA; MRI: magnetic resonance imaging; FET/PET: ^{18}Fluoro-*O*-(2) fluoroethyl-l-tyrosine/positron-emission-tomography; RT: radiotherapy; TMZ: Temozolomide.

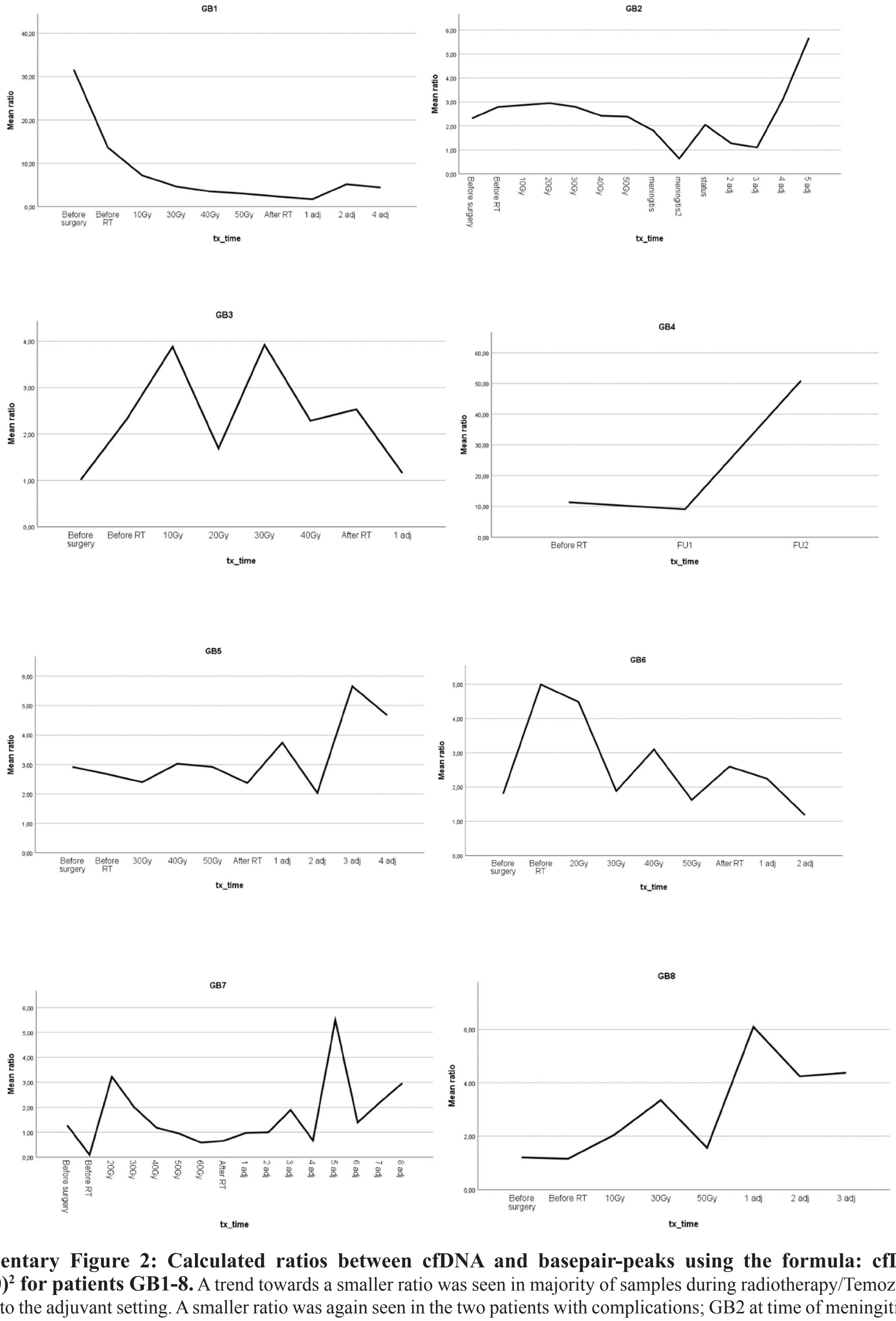

Supplementary Figure 2: Calculated ratios between cfDNA and basepair-peaks using the formula: cfDNA/(bp-peak/100)^2 for patients GB1-8. A trend towards a smaller ratio was seen in majority of samples during radiotherapy/Temozolomide as compared to the adjuvant setting. A smaller ratio was again seen in the two patients with complications; GB2 at time of meningitis and GB7 at time of intracerebral bleeding which took place during the sixth cycle of adjuvant treatment. The lesser ratio could illustrate a smaller fraction of ctDNA in the total cfDNA in these samples.

Supplementary Table 2: Cell-free DNA (cfDNA) concentration (ng/ml) with corresponding tumor size (mm^3)

cfDNA (ng/ml)	Tumor (mm^3)	cfDNA (ng/ml)	Tumor (mm^3)
0.3	1040	5.0	204
1.5	320	5.4	2340
1.7	320	5.7	352
2.4	756	5.9	736
2.6	324	6.6	104
3.0	1848	7.1	515
3.2	0	8.0	576
3.3	320	10.4	108
3.8	3243	10.8	3863
3.9	1699	11.1	0
3.9	2439	11.4	303
4.1	1334	12.0	0
4.4	1260	21.0	1911
4.5	243	26.4	0
4.8	0	63.0	870
4.8	0	73.4	1240

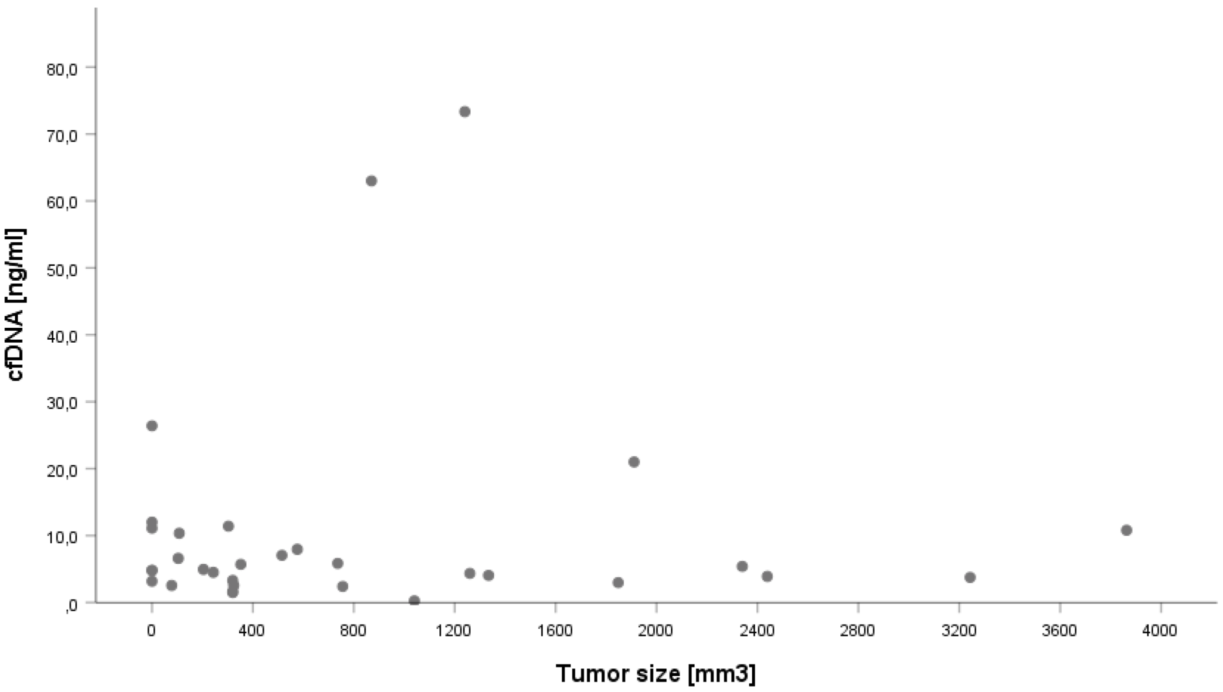

Supplementary Figure 3: Scatter plot with cell-free DNA (cfDNA) concentration (ng/ml) and tumor size (mm^3). We excluded one paired measurement during meningitis in GB2. No correlation was found using Spearman´s correlation analysis (Spearman´s rank correlation coefficient: 0.079; $p = 0.668$).
